# Expected and unexpected products of reactions of 2-hydrazinylbenzo­thia­zole with 3-nitro­benzene­sulfonyl chloride in different solvents

**DOI:** 10.1107/S2056989018005595

**Published:** 2018-04-17

**Authors:** Alexandra Morscher, Marcus V. N. de Souza, James L. Wardell, William T. A. Harrison

**Affiliations:** a Department of Chemistry, University of Aberdeen, Meston Walk, Aberdeen AB24 3UE, Scotland; b Instituto de Tecnologia em Fármacos – Farmanguinhos, Fiocruz. R. Sizenando, Nabuco, 100, Manguinhos, 21041-250, Rio de Janeiro, RJ, Brazil

**Keywords:** crystal structure, hydrazinyl­benzo­thiazole, hydrogen bonds, molecular salt

## Abstract

The syntheses and crystal structures of 2-[2-(propan-2-yl­idene)hydrazin­yl]-1,3-benzo­thia­zol-3-ium 3-nitro­benzene­sulfonate (C_10_H_12_N_2_S^+^·C_6_H_4_NO_5_S^−^), (I), 2-[2-(3-nitro­benzene­sulfon­yl)hydrazin­yl]-1,3-benzo­thia­zole (C_13_H_10_N_4_O_4_S_2_), (II) and 2-[2-(3-nitro­benzene­sulfon­yl)hydrazin­yl]-1,3-benzo­thia­zol-3-ium 3-nitro­benzene­sulfonate (C_13_H_11_N_4_O_4_S_2_^+^·C_6_H_4_NO_5_S^−^), (III) are reported. Salt (I) arose from an unexpected reaction of 2-hydrazinylbenzo­thia­zole with the acetone solvent in the presence of 3-nitro­benzene­sulfonyl chloride, whereas (II) and (III) were recovered from the equivalent reaction carried out in methanol. The crystal of (I) features ion pairs linked by pairs of N—H⋯O_s_ (s = sulfonate) hydrogen bonds; adjacent cations inter­act by way of short π–π stacking inter­actions between the thia­zole rings [centroid–centroid separation = 3.4274 (18) Å]. In (II), which crystallizes with two neutral mol­ecules in the asymmetric unit, the mol­ecules are linked by N—H⋯N and N—H⋯O_n_ (n = nitro) hydrogen bonds to generate [1] chains, which are cross-linked by C—H⋯O and π–π stacking inter­actions. The crystal of (III) features centrosymmetric tetra­mers (two cations and two anions) linked by cooperative N—H⋯O hydrogen bonds; C—H⋯O and π–π inter­actions occur between tetra­mers.

## Abstract 2

Two compounds arose from the same reaction in methanol and the other arose from an unexpected reaction with the acetone solvent.

## Chemical context   

Heteroaromatic benzo­thia­zole derivatives are well-studied compounds, due in the main to their various and useful biological activities (for a review, see Gulati *et al.*, 2017 ▸), but also to their fluorescent and optical properties (*e.g.* Liu *et al.*, 2018 ▸). Hydrazonyl derivatives, 2-Ar—CH=N—NH-benzo­thia­zoles, formed from 2-hydrazinylbenzo­thia­zole and ArCHO have attracted attention: for example, Katava *et al.* (2017 ▸) have reported anti­tumor activities and Behera & Manivannan (2017 ▸) studied their use as sensors. Less attention has been paid generally to 2-(ArSO_2_NHNH)-benzo­thia­zoles, although anti­microbial activities have been briefly reported (Rao *et al.*, 2005 ▸; Hipparagi *et al.*, 2007 ▸).

We have initiated a study of the syntheses, structures and biological activities of 2-(ArSO_2_NHNH)-benzo­thia­zoles and we now describe the structures of three products of the reactions of 2-hydrazinylbenzo­thia­zole with 3-nitro­benzene­sulfonyl chloride in different solvents, *viz*. 2-[2-(propan-2-yl­idene)hydrazin­yl]-1,3-benzo­thia­zol-3-ium 3-nitro­benz­enesulfonate (I), 2-[2-(3-nitro­benzene­sulfon­yl)hydrazin­yl]-1,3-benzo­thia­zole (II) and 2-[2-(3-nitro­benzene­sulfon­yl)hydrazin­yl]-1,3-benzo­thia­zol-3-ium 3-nitro­benzene­sulfonate (III).
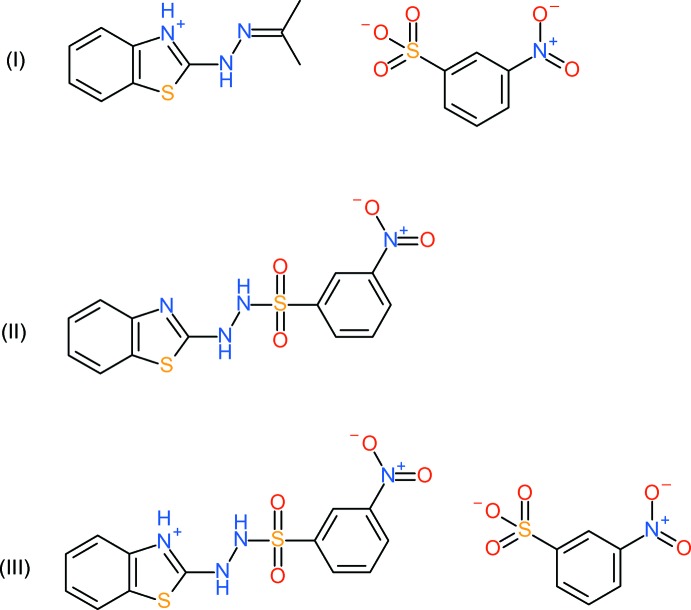


## Structural commentary   

**Figure 1:**
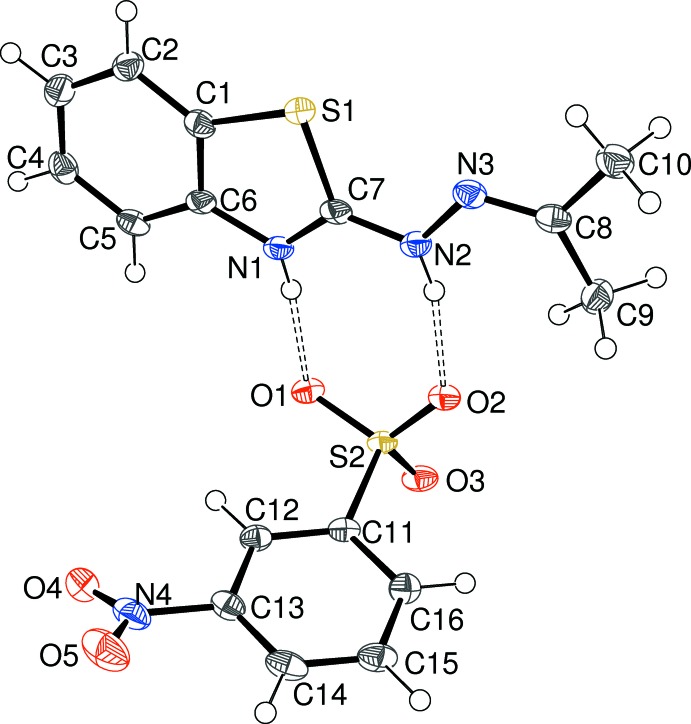
The asymmetric unit of (I) showing 50% displacement ellipsoids. Hydrogen bonds are indicated by double-dashed lines.

Compound (I) crystallizes in space group *P* 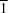 with one C_10_H_12_N_3_S^+^ cation (protonated at N1) and one C_6_H_4_NO_5_S^−^ sulfonate anion in the asymmetric unit (Fig. 1 ▸). Evidently, the starting hydrazone has reacted with the acetone solvent (Day & Whiting, 1970 ▸) to generate an *N*-propyl­idine group; at the same time, the sulfonyl chloride has been hydrolysed to sulfonic acid and a mol­ecular salt has crystallized after proton transfer from the sulfonic acid to the N atom of the thia­zole ring. The cation is close to planar; the dihedral angle between the benzo­thia­zole ring system (r.m.s. deviation = 0.005 Å) and the N2/N3/C8/C9/C10 grouping (r.m.s. deviation = 0.004 Å) is 7.89 (10)°; the C7—N2—N3—C8 torsion angle is −172.8 (2)°. The C8—N3 bond length of 1.278 (4) Å is fully consistent with double-bond character. In the anion, the nitro group is twisted by 26.7 (4)° with respect to the benzene ring. As expected, the S—O bond lengths of the sulfonate group are almost the same, indicating the usual delocalization of the negative charge and the same situation is found in compound (III) described below.

**Figure 2:**
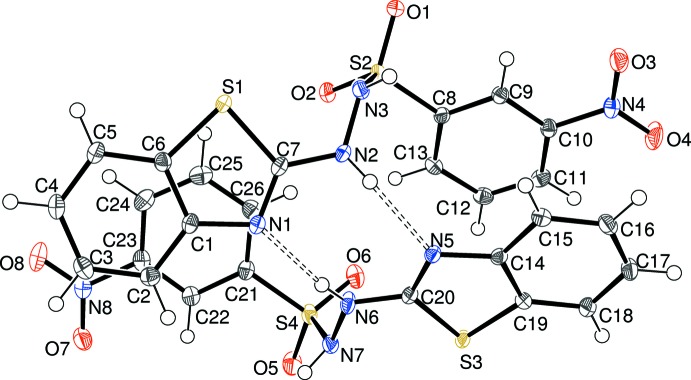
The asymmetric unit of (II) showing 50% displacement ellipsoids. Hydrogen bonds are indicated by double-dashed lines.

Compound (II) represents the expected condensation product of the starting hydrazone and sulfonyl chloride and crystallizes with two neutral C_13_H_10_N_4_O_4_S mol­ecules in the asymmetric unit (Fig. 2 ▸) in space group *P* 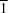. In the first (S1) mol­ecule, the dihedral angle between the benzo­thia­zole ring system (r.m.s. deviation = 0.013 Å) and the C8 benzene ring is 32.59 (4)°; the nitro group is twisted by 0.68 (7)° from the C8 benzene ring. The C7—N2—N3—S2 torsion angle is −99.88 (12) and the H2—N2—N3—H3 torsion angle is −54 (2)°. The bond-angle sum at N2 is 359.9°, indicative of *sp* ^2^ hybridization, whereas the corresponding value for N3 of 341.1° points towards substantial *sp* ^3^ hybrid character. The C7—N2 bond length of 1.3529 (16) Å is short for a nominal single bond, presumably indicative of conjugation of the N2 nominal lone pair of electrons with the adjacent ring system. In the second (S3) mol­ecule, the corresponding geometrical data are 0.008 Å (r.m.s. deviation for S3 ring system), 30.01 (3)° (S3/C21 rings), 3.46 (13)° (nitro group and C21 ring), −103.53 (12)° (C20—N6—N7—S4), −50.3 (18)° (H6—N6—N7—H7), 359.9° (bond-angle sum at N6), 341.7° (bond-angle sum at N7) and 1.3549 (16) Å (C20—N6 bond length). All-in-all, the S1 and S3 mol­ecules have similar conformations as indicated by the r.m.s. overlay fit of 0.221 Å for their non-hydrogen atoms.

**Figure 3:**
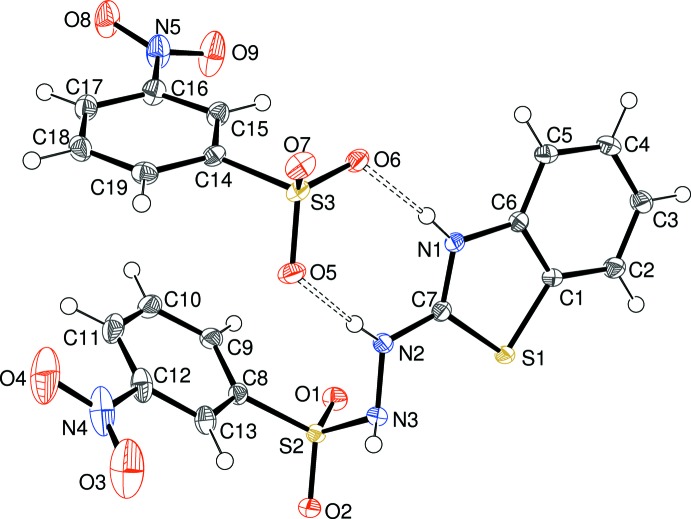
The asymmetric unit of (III) showing 50% displacement ellipsoids. Hydrogen bonds are indicated by double-dashed lines.

Compound (III), which was recovered from the same reaction as (II), represents the same condensation product, which has gone on to further react with a hydrolysed sulfonyl chloride species to form a mol­ecular salt (proton transfer to N1). Once again, the space group is *P* 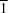 and one cation and one anion (Fig. 3 ▸) make up the asymmetric unit. The benzo­thia­zole ring system (r.m.s. deviation = 0.005 Å) subtends a dihedral angle of 57.54 (3)° with the C8 benzene ring and the nitro group is twisted from its attached ring by 4.8 (3)°. The C7—N2—N3—S2 and H2—N2—N3—H3 torsion angles are −110.54 (12) and −48.5 (19)°, respectively. The bond-angle sums at N2 and N3 are 359.0 and 339.1°, respectively, and the same conclusions *re* hybridization states for these atoms as in (II) may be drawn. This is backed up by the shortened C7—N2 bond length of 1.3317 (17) Å in (III) compared to (II). presumably because resonance is enhanced by the positive charge on N1. In the anion, the nitro group is twisted from its attached ring by 17.7 (2)°.

## Supra­molecular features   

**Figure 4:**
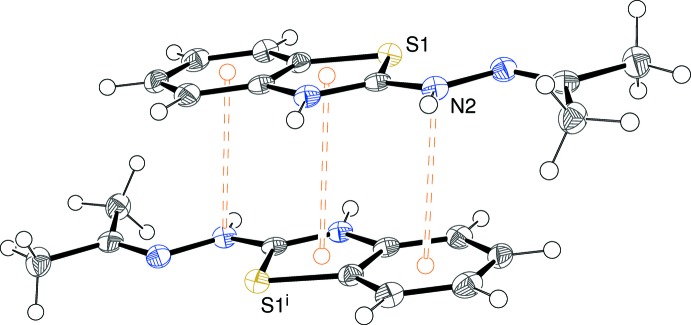
Detail of the extended structure of (I) showing π–π stacking between inversion-related thia­zole rings and possible secondary N⋯π inter­actions. Symmetry code: (i) −x, 1 − y, 1 − z.

In the crystal of (I), the cation and the anion are linked by a pair of N—H⋯O hydrogen bonds (Table 1 ▸), which generate an 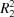
(8) loop. The ion pairs are connected by various weak C—H⋯O inter­actions, with the acceptor O atoms being parts of the sulfonate and nitro groups. No C—H⋯π inter­actions could be identified in the crystal of (I) but aromatic π–π stacking inter­actions are seen, with the shortest centroid–centroid separation of 3.4274 (18) Å (slippage = 0.729 Å) occurring between inversion-related pairs of thia­zole rings (Fig. 4 ▸); atom N2 of the hydrazone group lies above the benzene ring (*Cg*⋯N2 = 3.385 Å) and possibly provides some additional stabilization. Taken together, the directional inter­molecular inter­actions in (I) generate a three-dimensional network.

**Figure 5:**
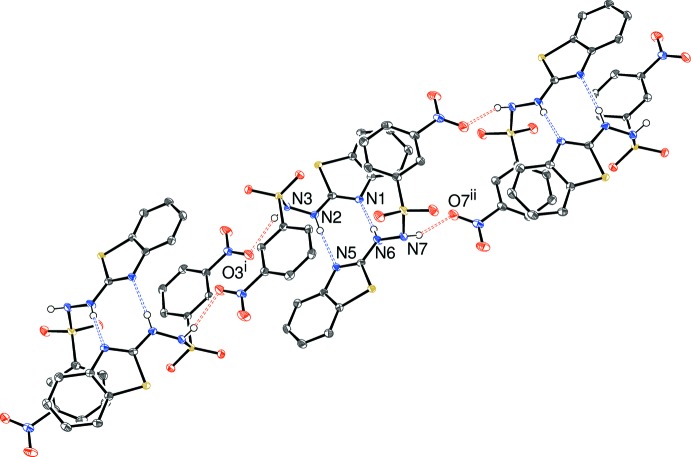
Fragment of a [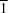
1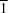] hydrogen-bonded chain in (II) with the N—H⋯N and N—H⋯O bonds shown as blue and red double-dashed lines, respectively. All C-bonded hydrogen atoms have been omitted for clarity. Symmetry codes: (i) −*x*, 1 − *y*, −z; (ii) 1 − *x*, −*y*, 1 − *z*.

The dominant inter­molecular inter­actions in (II) are N—H⋯N and N—H⋯O hydrogen bonds (Table 2 ▸); the first of these (N2—H2⋯N5 and N6—H6⋯N1) occur in the arbitrarily chosen asymmetric unit to link the mol­ecules into dimers that ‘slot together’: the dihedral angle between the benzo­thia­zole planes in the two mol­ecules is 36.06 (4)° and the pendant benzene sulfonyl groups project to the same side of the ensemble. The N—H⋯O_n_ (n = nitro) links connect the dimers into infinite [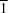
1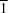] chains (Fig. 5 ▸). A number of weak C—H⋯O inter­actions are also observed, which serve to cross-link the chains. Several π–π stacking contacts occur in the crystal of (II), with the shortest [centroid–centroid separation = 3.5186 (7)Å] occurring between the C8–C13 and C14–C19 rings. Finally, a short N8—O7⋯π (π = centroid of the C21–C26 benzene ring) contact is observed with N⋯π = 3.2497 (12) Å and N—O⋯π = 86.24 (8)°.

**Figure 6:**
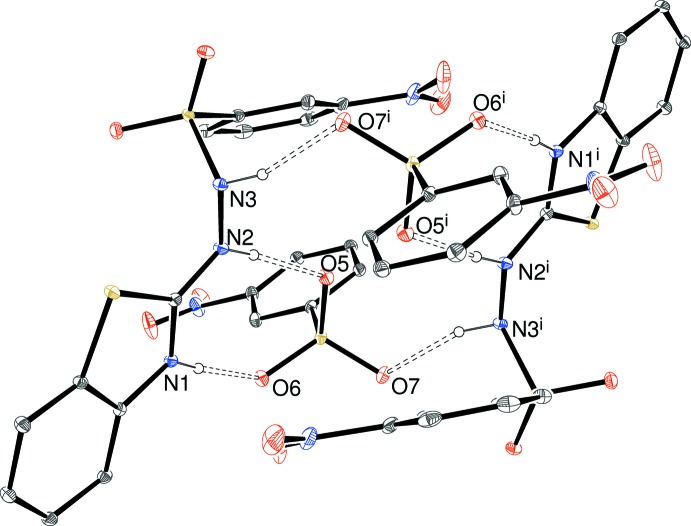
An inversion-generated tetra­mer in the crystal of (III). Symmetry code: (i) 1 − *x*, 1 − *y*, −*z*.

The packing in (III) features a pair of cation-to-anion N—H⋯O links from N1 and N2 (Table 3 ▸), which is essentially the same motif as seen in (I). The N3—H3 grouping links to a symmetry-generated sulfonate O atom and a centrosymmetric tetra­mer (two cations and two anions) results (Fig. 6 ▸). A pair of weak C—H⋯O inter­actions helps to provide cohesion between tetra­mers in the crystal and π–π stacking is also observed, with the shortest centroid–centroid separation being 3.6743 (8) Å between the thia­zole and C1–C6 rings.

## Database survey   

A survey of of the Cambridge Structural Database (Groom *et al.*, 2016 ▸: updated to March 2018) for benzo­thia­zole hydrazones revealed the prototype compound, benzo­thia­zol-2-yl-hydrazine (refcode: NAPKAR; Rajnikant *et al.*, 2005 ▸) as well as three derivatives with various substituents attached to the benzene ring, *viz*. EVARID (Liu *et al.*, 2011 ▸), LAPCAI (Fun *et al.*, 2012*a* ▸) and LEFTEX (Fun *et al.*, 2012*b* ▸). No hits for benzene­sulfonyl­hydrazino-benzo­thia­zoles were recorded.

## Synthesis and crystallization   

To prepare (I), a mixture of 2-hydrazinylbenzo­thia­zole (1.00 mmol) and 3-nitro­benzene­sulfonyl chloride (1.00 mmol) in acetone (15 ml) was gently heated at 313–323 K for 30 minutes, then rotary evaporated and the residue was recrystallized by slow evaporation from methanol solution at room temperature; m.p. 444–445 K. ESI–HRMS (*M* + H). Calculated: 206.0752 for C_10_H_12_N_3_S, found: 206.0755. IR: 2930(br), 1621, 1530, 1350, 1241, 1151, 1028 cm^−1^

Compounds (II) and (III) arose from the same reaction: a solution of 2-hydrazinylbenzo­thia­zole (1.00 mmol) and 3-nitro­benzene­sulfonyl chloride (1.00 mmol) in methanol (15 ml) was gently heated at 313–323 K for 30 minutes, then rotary evaporated and the residue was recrystallized by slow evaporation from methanol solution at room temperature. A mixture of two distinct crystalline products, one yellow [compound (II)] and the other colourless [compound (III)], was isolated. These were separated by eye, and each product was futher recrystallized from methanol solution. (II); m.p. 442–444 K. ESI–HRMS (*M* − H). Calculated: 349.0222 for C_13_H_9_N_4_O_2_S_2_: found: 351.0220 ESI–HRMS (*M* + H). Calculated: 351.0065 for C_13_H_11_N_4_O_2_S_2_: found: 349.0062 IR; 2989 (*br*), 1531, 1457, 1341, 1306, 1167 cm^−1^. (III): m.p. 463–466 K. ESI–HRMS (*M* + H). Calculated: 351.0065 for C_13_H_11_N_4_O_2_S_2_: found: 349.0065 IR: 2870 (*br*), 1553, 1436, 1363, 1241, 1127, 1065 cm^−1^.

## Refinement   

Crystal data, data collection and structure refinement details are summarized in Table 4 ▸. The N-bound hydrogen atoms were located in difference maps and their positions freely refined. The C-bound hydrogen atoms were geometrically placed (C—H = 0.95–0.98 Å) and refined as riding atoms. The constraint *U*_iso_(H) = 1.2*U*_eq_(carrier) or 1.5*U*_eq_(methyl carrier) was applied in all cases. The methyl groups in (I) were allowed to rotate, but not to tip, to best fit the electron density.

## Supplementary Material

Crystal structure: contains datablock(s) I, II, III, global. DOI: 10.1107/S2056989018005595/sj5555sup1.cif

Structure factors: contains datablock(s) I. DOI: 10.1107/S2056989018005595/sj5555Isup2.hkl

Structure factors: contains datablock(s) II. DOI: 10.1107/S2056989018005595/sj5555IIsup3.hkl

Structure factors: contains datablock(s) III. DOI: 10.1107/S2056989018005595/sj5555IIIsup4.hkl

Supporting information file. DOI: 10.1107/S2056989018005595/sj5555Isup5.cml

Supporting information file. DOI: 10.1107/S2056989018005595/sj5555IIsup6.cml

Supporting information file. DOI: 10.1107/S2056989018005595/sj5555IIIsup7.cml

CCDC references: 1835988, 1835987, 1835986

Additional supporting information:  crystallographic information; 3D view; checkCIF report

## Figures and Tables

**Table 1:** Hydrogen-bond geometry (Å, °) for (I)

*D*—H⋯*A*	*D*—H	H⋯*A*	*D*⋯*A*	*D*—H⋯*A*
N1—H1⋯O1	0.86 (4)	1.87 (4)	2.721 (3)	167 (3)
N2—H2⋯O2	0.82 (4)	1.96 (4)	2.773 (3)	169 (3)
C3—H3⋯O2^i^	0.95	2.51	3.415 (4)	160
C4—H4⋯O4^ii^	0.95	2.55	3.292 (4)	135
C10—H10*B*⋯O4^iii^	0.98	2.54	3.297 (5)	134
C15—H15⋯O3^iv^	0.95	2.50	3.315 (4)	144

Symmetry codes: (i) 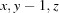; (ii) 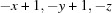; (iii) 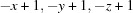; (iv) 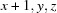.

**Table 2:** Hydrogen-bond geometry (Å, °) for (II)

*D*—H⋯*A*	*D*—H	H⋯*A*	*D*⋯*A*	*D*—H⋯*A*
N2—H2⋯N5	0.816 (18)	2.033 (18)	2.8447 (15)	172.7 (16)
N3—H3⋯O3^i^	0.848 (17)	2.129 (18)	2.9427 (15)	160.8 (15)
N6—H6⋯N1	0.820 (18)	2.050 (18)	2.8601 (15)	169.2 (17)
N7—H7⋯O7^ii^	0.871 (17)	2.123 (18)	2.9472 (15)	157.6 (15)
C15—H15⋯O3^i^	0.95	2.65	3.4888 (17)	147
C26—H26⋯O2	0.95	2.44	3.1774 (16)	134
C5—H5⋯O1^iii^	0.95	2.66	3.3218 (16)	127
C13—H13⋯O6	0.95	2.56	3.2731 (16)	133

Symmetry codes: (i) 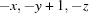; (ii) 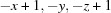; (iii) 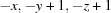.

**Table 3:** Hydrogen-bond geometry (Å, °) for (III)

*D*—H⋯*A*	*D*—H	H⋯*A*	*D*⋯*A*	*D*—H⋯*A*
N1—H1⋯O6	0.841 (19)	1.888 (19)	2.7267 (15)	175.2 (17)
N2—H2⋯O5	0.826 (19)	1.92 (2)	2.7489 (16)	175.4 (18)
N3—H3⋯O7^i^	0.869 (18)	1.968 (19)	2.8058 (16)	161.6 (16)
C2—H2*A*⋯O7^ii^	0.95	2.55	3.2510 (18)	130
C9—H9⋯O8^iii^	0.95	2.58	3.487 (2)	161

Symmetry codes: (i) 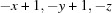; (ii) 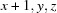; (iii) 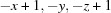.

**Table 4:** Experimental details

	(I)	(II)	(III)
Crystal data			
Chemical formula	C_10_H_12_N_3_S^+^·C_6_H_4_NO_5_S^−^	C_13_H_10_N_4_O_4_S_2_	C_13_H_11_N_4_O_4_S_2_^+^·C_6_H_4_NO_5_S^−^
*M*_r_	408.45	350.37	553.54
Crystal system, space group	Triclinic, *P* 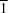	Triclinic, *P* 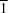	Triclinic, *P* 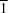
Temperature (K)	100	100	100
*a*, *b*, *c* (Å)	7.5308 (4), 10.9167 (7), 12.4438 (8)	6.83537 (15), 13.5788 (3), 15.6907 (4)	10.0399 (5), 10.7585 (4), 11.3372 (6)
α, β, γ (°)	66.058 (6), 79.034 (5), 72.156 (5)	99.382 (2), 98.2324 (19), 91.9841 (19)	85.607 (4), 71.369 (5), 77.115 (4)
*V* (Å^3^)	887.47 (10)	1419.55 (6)	1131.16 (10)
*Z*	2	4	2
Radiation type	Mo *K*α	Mo *K*α	Mo *K*α
μ (mm^−1^)	0.34	0.40	0.39
Crystal size (mm)	0.06 × 0.05 × 0.01	0.15 × 0.10 × 0.03	0.23 × 0.18 × 0.04

Data collection			
Diffractometer	Rigaku Mercury CCD	Rigaku Mercury CCD	Rigaku Mercury CCD
Absorption correction	Multi-scan (*FS_ABSCOR*; Rigaku, 2013 ▸)	Multi-scan (*FS_ABSCOR*; Rigaku, 2013 ▸)	Multi-scan (*FS_ABSCOR*; Rigaku, 2013 ▸)
*T*_min_, *T*_max_	0.614, 1.000	0.861, 1.000	0.879, 1.000
No. of measured, independent and observed [*I* > 2σ(*I*)] reflections	11341, 3490, 3249	24032, 6465, 6033	19781, 5159, 4880
*R*_int_	0.036	0.018	0.017
(sin θ/λ)_max_ (Å^−1^)	0.617	0.649	0.651

Refinement			
*R*[*F* ^2^ > 2σ(*F* ^2^)], *wR*(*F* ^2^), *S*	0.066, 0.184, 1.11	0.027, 0.072, 1.04	0.029, 0.076, 1.02
No. of reflections	3490	6465	5159
No. of parameters	250	427	334
H-atom treatment	H atoms treated by a mixture of independent and constrained refinement	H atoms treated by a mixture of independent and constrained refinement	H atoms treated by a mixture of independent and constrained refinement
Δρ_max_, Δρ_min_ (e Å^−3^)	1.32, −0.63	0.40, −0.38	0.39, −0.42

Computer programs: *CrystalClear* (Rigaku, 2012 ▸), *SHELXS97* (Sheldrick, 2008 ▸), *SHELXL2014* (Sheldrick, 2015 ▸), *ORTEP-3 for Windows* (Farrugia, 2012 ▸) and *publCIF* (Westrip, 2010 ▸).

## supplementary crystallographic information

### 2-[2-(Propan-2-ylidene)hydrazinyl]-1,3-benzothiazol-3-ium 3-nitrobenzenesulfonate (I) Crystal data

**Table:**

C_10_H_12_N_3_S^+^·C_6_H_4_NO_5_S^−^	*Z* = 2
*M**_r_* = 408.45	*F*(000) = 424
Triclinic, *P*1	*D*_x_ = 1.528 Mg m^−^^3^
*a* = 7.5308 (4) Å	Mo *K*α radiation, λ = 0.71075 Å
*b* = 10.9167 (7) Å	Cell parameters from 6372 reflections
*c* = 12.4438 (8) Å	θ = 2.8–30.4°
α = 66.058 (6)°	µ = 0.34 mm^−^^1^
β = 79.034 (5)°	*T* = 100 K
γ = 72.156 (5)°	Block, colourless
*V* = 887.47 (10) Å^3^	0.06 × 0.05 × 0.01 mm

### 2-[2-(Propan-2-ylidene)hydrazinyl]-1,3-benzothiazol-3-ium 3-nitrobenzenesulfonate (I) Data collection

**Table:**

Rigaku Mercury CCD diffractometer	3249 reflections with *I* > 2σ(*I*)
ω scans	*R*_int_ = 0.036
Absorption correction: multi-scan (*FS_ABSCOR*; Rigaku, 2013)	θ_max_ = 26.0°, θ_min_ = 2.9°
*T*_min_ = 0.614, *T*_max_ = 1.000	*h* = −9→7
11341 measured reflections	*k* = −13→13
3490 independent reflections	*l* = −15→15

### 2-[2-(Propan-2-ylidene)hydrazinyl]-1,3-benzothiazol-3-ium 3-nitrobenzenesulfonate (I) Refinement

**Table:**

Refinement on *F*^2^	Primary atom site location: structure-invariant direct methods
Least-squares matrix: full	Hydrogen site location: mixed
*R*[*F*^2^ > 2σ(*F*^2^)] = 0.066	H atoms treated by a mixture of independent and constrained refinement
*wR*(*F*^2^) = 0.184	*w* = 1/[σ^2^(*F*_o_^2^) + (0.1206*P*)^2^ + 0.8477*P*] where *P* = (*F*_o_^2^ + 2*F*_c_^2^)/3
*S* = 1.11	(Δ/σ)_max_ = 0.001
3490 reflections	Δρ_max_ = 1.32 e Å^−^^3^
250 parameters	Δρ_min_ = −0.63 e Å^−^^3^
0 restraints	

### 2-[2-(Propan-2-ylidene)hydrazinyl]-1,3-benzothiazol-3-ium 3-nitrobenzenesulfonate (I) Special details

**Table:**

Geometry. All esds (except the esd in the dihedral angle between two l.s. planes) are estimated using the full covariance matrix. The cell esds are taken into account individually in the estimation of esds in distances, angles and torsion angles; correlations between esds in cell parameters are only used when they are defined by crystal symmetry. An approximate (isotropic) treatment of cell esds is used for estimating esds involving l.s. planes.

### 2-[2-(Propan-2-ylidene)hydrazinyl]-1,3-benzothiazol-3-ium 3-nitrobenzenesulfonate (I) Fractional atomic coordinates and isotropic or equivalent isotropic displacement parameters (Å^2^)

**Table:**

	*x*	*y*	*z*	*U*_iso_*/*U*_eq_	
C1	0.2281 (4)	0.3162 (3)	0.5033 (3)	0.0203 (6)	
C2	0.2122 (4)	0.1899 (3)	0.5087 (3)	0.0228 (6)	
H2A	0.2284	0.1105	0.5798	0.027*	
C3	0.1720 (4)	0.1837 (3)	0.4070 (3)	0.0262 (7)	
H3	0.1611	0.0986	0.4083	0.031*	
C4	0.1472 (4)	0.3006 (3)	0.3030 (3)	0.0248 (6)	
H4	0.1204	0.2937	0.2344	0.030*	
C5	0.1609 (4)	0.4267 (3)	0.2978 (3)	0.0231 (6)	
H5	0.1419	0.5065	0.2270	0.028*	
C6	0.2032 (4)	0.4330 (3)	0.3988 (3)	0.0191 (6)	
C7	0.2606 (4)	0.5238 (3)	0.5207 (3)	0.0187 (6)	
C8	0.3338 (4)	0.6563 (3)	0.7133 (3)	0.0228 (6)	
C9	0.2989 (5)	0.8099 (3)	0.6481 (3)	0.0276 (7)	
H9A	0.3122	0.8531	0.7004	0.041*	
H9B	0.3897	0.8293	0.5795	0.041*	
H9C	0.1719	0.8476	0.6217	0.041*	
C10	0.3777 (5)	0.6021 (4)	0.8395 (3)	0.0298 (7)	
H10A	0.3780	0.6793	0.8606	0.045*	
H10B	0.2827	0.5556	0.8906	0.045*	
H10C	0.5011	0.5359	0.8499	0.045*	
N1	0.2209 (3)	0.5486 (3)	0.4133 (2)	0.0190 (5)	
H1	0.188 (5)	0.633 (4)	0.364 (3)	0.023*	
N2	0.2853 (3)	0.6164 (3)	0.5555 (2)	0.0197 (5)	
H2	0.272 (5)	0.697 (4)	0.509 (3)	0.024*	
N3	0.3282 (3)	0.5676 (3)	0.6723 (2)	0.0221 (5)	
S1	0.27708 (10)	0.35384 (7)	0.61732 (6)	0.0196 (2)	
C11	0.4451 (4)	0.9109 (3)	0.1794 (2)	0.0183 (6)	
C12	0.5278 (4)	0.8235 (3)	0.1184 (2)	0.0195 (6)	
H12	0.4602	0.7699	0.1067	0.023*	
C13	0.7139 (4)	0.8170 (3)	0.0747 (3)	0.0223 (6)	
C14	0.8145 (4)	0.8949 (3)	0.0890 (3)	0.0262 (7)	
H14	0.9422	0.8861	0.0602	0.031*	
C15	0.7274 (4)	0.9858 (3)	0.1455 (3)	0.0259 (7)	
H15	0.7938	1.0430	0.1529	0.031*	
C16	0.5418 (4)	0.9941 (3)	0.1918 (3)	0.0221 (6)	
H16	0.4820	1.0560	0.2315	0.027*	
S2	0.21789 (9)	0.91074 (7)	0.25157 (6)	0.0184 (2)	
N4	0.8044 (4)	0.7214 (3)	0.0138 (2)	0.0302 (6)	
O1	0.1647 (3)	0.8018 (2)	0.23498 (18)	0.0222 (5)	
O2	0.2432 (3)	0.8753 (2)	0.37542 (18)	0.0238 (5)	
O3	0.1002 (3)	1.0479 (2)	0.19792 (19)	0.0255 (5)	
O4	0.7054 (4)	0.6912 (3)	−0.0342 (2)	0.0360 (6)	
O5	0.9750 (3)	0.6763 (3)	0.0148 (2)	0.0432 (7)	

### 2-[2-(Propan-2-ylidene)hydrazinyl]-1,3-benzothiazol-3-ium 3-nitrobenzenesulfonate (I) Atomic displacement parameters (Å^2^)

**Table:**

	*U*^11^	*U*^22^	*U*^33^	*U*^12^	*U*^13^	*U*^23^
C1	0.0162 (13)	0.0222 (14)	0.0203 (14)	−0.0039 (11)	0.0017 (11)	−0.0077 (11)
C2	0.0213 (14)	0.0197 (14)	0.0270 (15)	−0.0063 (11)	0.0015 (12)	−0.0089 (12)
C3	0.0203 (14)	0.0263 (15)	0.0358 (17)	−0.0088 (12)	0.0037 (13)	−0.0158 (14)
C4	0.0183 (14)	0.0342 (16)	0.0259 (15)	−0.0076 (12)	0.0028 (12)	−0.0164 (13)
C5	0.0171 (13)	0.0290 (15)	0.0204 (14)	−0.0085 (12)	0.0039 (11)	−0.0069 (12)
C6	0.0122 (12)	0.0201 (13)	0.0227 (14)	−0.0056 (10)	0.0048 (11)	−0.0073 (11)
C7	0.0099 (12)	0.0218 (14)	0.0226 (14)	−0.0058 (10)	0.0047 (10)	−0.0078 (11)
C8	0.0135 (13)	0.0302 (16)	0.0254 (15)	−0.0100 (12)	0.0056 (11)	−0.0107 (13)
C9	0.0294 (16)	0.0281 (16)	0.0300 (16)	−0.0103 (13)	−0.0003 (13)	−0.0140 (13)
C10	0.0295 (16)	0.0390 (18)	0.0248 (16)	−0.0160 (14)	0.0026 (13)	−0.0125 (14)
N1	0.0162 (11)	0.0178 (12)	0.0196 (12)	−0.0062 (9)	0.0028 (9)	−0.0042 (10)
N2	0.0188 (12)	0.0178 (12)	0.0214 (12)	−0.0070 (10)	0.0009 (10)	−0.0056 (10)
N3	0.0178 (12)	0.0264 (13)	0.0220 (12)	−0.0088 (10)	0.0023 (10)	−0.0084 (10)
S1	0.0197 (4)	0.0179 (4)	0.0193 (4)	−0.0062 (3)	−0.0008 (3)	−0.0044 (3)
C11	0.0153 (13)	0.0199 (13)	0.0170 (13)	−0.0062 (11)	−0.0021 (10)	−0.0026 (11)
C12	0.0202 (14)	0.0197 (13)	0.0168 (13)	−0.0071 (11)	−0.0021 (11)	−0.0034 (11)
C13	0.0201 (14)	0.0241 (14)	0.0174 (13)	−0.0032 (12)	−0.0003 (11)	−0.0045 (11)
C14	0.0156 (13)	0.0353 (17)	0.0207 (14)	−0.0068 (12)	−0.0018 (11)	−0.0029 (12)
C15	0.0177 (14)	0.0363 (17)	0.0250 (15)	−0.0141 (13)	−0.0015 (12)	−0.0077 (13)
C16	0.0217 (14)	0.0231 (14)	0.0213 (14)	−0.0066 (12)	−0.0024 (11)	−0.0071 (12)
S2	0.0147 (4)	0.0195 (4)	0.0200 (4)	−0.0077 (3)	0.0015 (3)	−0.0051 (3)
N4	0.0333 (15)	0.0265 (14)	0.0196 (13)	−0.0047 (12)	0.0065 (11)	−0.0033 (11)
O1	0.0214 (10)	0.0224 (10)	0.0223 (10)	−0.0098 (8)	−0.0013 (8)	−0.0049 (8)
O2	0.0250 (11)	0.0247 (11)	0.0221 (11)	−0.0119 (9)	0.0029 (8)	−0.0071 (8)
O3	0.0174 (10)	0.0240 (11)	0.0305 (12)	−0.0078 (9)	0.0016 (9)	−0.0052 (9)
O4	0.0485 (15)	0.0343 (13)	0.0270 (12)	−0.0169 (11)	0.0107 (11)	−0.0144 (10)
O5	0.0280 (13)	0.0419 (14)	0.0406 (15)	0.0041 (11)	0.0079 (11)	−0.0105 (12)

### 2-[2-(Propan-2-ylidene)hydrazinyl]-1,3-benzothiazol-3-ium 3-nitrobenzenesulfonate (I) Geometric parameters (Å, º)

**Table:**

C1—C2	1.393 (4)	C10—H10B	0.9800
C1—C6	1.397 (4)	C10—H10C	0.9800
C1—S1	1.757 (3)	N1—H1	0.86 (4)
C2—C3	1.388 (4)	N2—N3	1.395 (3)
C2—H2A	0.9500	N2—H2	0.82 (4)
C3—C4	1.394 (5)	C11—C12	1.386 (4)
C3—H3	0.9500	C11—C16	1.393 (4)
C4—C5	1.387 (4)	C11—S2	1.775 (3)
C4—H4	0.9500	C12—C13	1.395 (4)
C5—C6	1.386 (4)	C12—H12	0.9500
C5—H5	0.9500	C13—C14	1.377 (5)
C6—N1	1.393 (4)	C13—N4	1.462 (4)
C7—N2	1.321 (4)	C14—C15	1.379 (5)
C7—N1	1.327 (4)	C14—H14	0.9500
C7—S1	1.734 (3)	C15—C16	1.398 (4)
C8—N3	1.278 (4)	C15—H15	0.9500
C8—C9	1.499 (4)	C16—H16	0.9500
C8—C10	1.500 (4)	S2—O3	1.440 (2)
C9—H9A	0.9800	S2—O1	1.463 (2)
C9—H9B	0.9800	S2—O2	1.464 (2)
C9—H9C	0.9800	N4—O4	1.226 (4)
C10—H10A	0.9800	N4—O5	1.227 (4)
			
C2—C1—C6	120.9 (3)	C7—N1—C6	114.0 (2)
C2—C1—S1	127.7 (2)	C7—N1—H1	120 (2)
C6—C1—S1	111.4 (2)	C6—N1—H1	125 (2)
C3—C2—C1	117.9 (3)	C7—N2—N3	115.8 (2)
C3—C2—H2A	121.0	C7—N2—H2	119 (2)
C1—C2—H2A	121.0	N3—N2—H2	125 (2)
C2—C3—C4	120.9 (3)	C8—N3—N2	117.8 (3)
C2—C3—H3	119.6	C7—S1—C1	89.17 (14)
C4—C3—H3	119.6	C12—C11—C16	121.1 (3)
C5—C4—C3	121.3 (3)	C12—C11—S2	120.3 (2)
C5—C4—H4	119.4	C16—C11—S2	118.5 (2)
C3—C4—H4	119.4	C11—C12—C13	117.6 (3)
C6—C5—C4	118.0 (3)	C11—C12—H12	121.2
C6—C5—H5	121.0	C13—C12—H12	121.2
C4—C5—H5	121.0	C14—C13—C12	122.4 (3)
C5—C6—N1	127.4 (3)	C14—C13—N4	119.5 (3)
C5—C6—C1	120.9 (3)	C12—C13—N4	118.0 (3)
N1—C6—C1	111.6 (3)	C13—C14—C15	119.1 (3)
N2—C7—N1	125.2 (3)	C13—C14—H14	120.4
N2—C7—S1	121.0 (2)	C15—C14—H14	120.4
N1—C7—S1	113.8 (2)	C14—C15—C16	120.1 (3)
N3—C8—C9	126.5 (3)	C14—C15—H15	119.9
N3—C8—C10	117.1 (3)	C16—C15—H15	119.9
C9—C8—C10	116.5 (3)	C11—C16—C15	119.5 (3)
C8—C9—H9A	109.5	C11—C16—H16	120.3
C8—C9—H9B	109.5	C15—C16—H16	120.3
H9A—C9—H9B	109.5	O3—S2—O1	114.29 (12)
C8—C9—H9C	109.5	O3—S2—O2	113.10 (13)
H9A—C9—H9C	109.5	O1—S2—O2	111.73 (12)
H9B—C9—H9C	109.5	O3—S2—C11	106.88 (13)
C8—C10—H10A	109.5	O1—S2—C11	105.44 (13)
C8—C10—H10B	109.5	O2—S2—C11	104.46 (12)
H10A—C10—H10B	109.5	O4—N4—O5	124.4 (3)
C8—C10—H10C	109.5	O4—N4—C13	118.1 (3)
H10A—C10—H10C	109.5	O5—N4—C13	117.5 (3)
H10B—C10—H10C	109.5		
			
C6—C1—C2—C3	0.4 (4)	C2—C1—S1—C7	−179.2 (3)
S1—C1—C2—C3	179.9 (2)	C6—C1—S1—C7	0.4 (2)
C1—C2—C3—C4	−0.4 (4)	C16—C11—C12—C13	3.0 (4)
C2—C3—C4—C5	−0.3 (4)	S2—C11—C12—C13	−173.0 (2)
C3—C4—C5—C6	1.0 (4)	C11—C12—C13—C14	−0.9 (4)
C4—C5—C6—N1	−179.0 (3)	C11—C12—C13—N4	177.9 (2)
C4—C5—C6—C1	−1.0 (4)	C12—C13—C14—C15	−1.9 (4)
C2—C1—C6—C5	0.4 (4)	N4—C13—C14—C15	179.3 (3)
S1—C1—C6—C5	−179.2 (2)	C13—C14—C15—C16	2.6 (4)
C2—C1—C6—N1	178.6 (2)	C12—C11—C16—C15	−2.2 (4)
S1—C1—C6—N1	−0.9 (3)	S2—C11—C16—C15	173.8 (2)
N2—C7—N1—C6	179.5 (2)	C14—C15—C16—C11	−0.6 (4)
S1—C7—N1—C6	−0.9 (3)	C12—C11—S2—O3	−118.7 (2)
C5—C6—N1—C7	179.3 (3)	C16—C11—S2—O3	65.2 (2)
C1—C6—N1—C7	1.2 (3)	C12—C11—S2—O1	3.3 (3)
N1—C7—N2—N3	−179.3 (2)	C16—C11—S2—O1	−172.8 (2)
S1—C7—N2—N3	1.2 (3)	C12—C11—S2—O2	121.2 (2)
C9—C8—N3—N2	−0.1 (4)	C16—C11—S2—O2	−54.9 (2)
C10—C8—N3—N2	179.2 (2)	C14—C13—N4—O4	−154.8 (3)
C7—N2—N3—C8	−172.8 (2)	C12—C13—N4—O4	26.4 (4)
N2—C7—S1—C1	179.9 (2)	C14—C13—N4—O5	25.7 (4)
N1—C7—S1—C1	0.3 (2)	C12—C13—N4—O5	−153.2 (3)

### 2-[2-(Propan-2-ylidene)hydrazinyl]-1,3-benzothiazol-3-ium 3-nitrobenzenesulfonate (I) Hydrogen-bond geometry (Å, º)

**Table:**

*D*—H···*A*	*D*—H	H···*A*	*D*···*A*	*D*—H···*A*
N1—H1···O1	0.86 (4)	1.87 (4)	2.721 (3)	167 (3)
N2—H2···O2	0.82 (4)	1.96 (4)	2.773 (3)	169 (3)
C3—H3···O2^i^	0.95	2.51	3.415 (4)	160
C4—H4···O4^ii^	0.95	2.55	3.292 (4)	135
C10—H10*B*···O4^iii^	0.98	2.54	3.297 (5)	134
C15—H15···O3^iv^	0.95	2.50	3.315 (4)	144

Symmetry codes: (i) *x*, *y*−1, *z*; (ii) −*x*+1, −*y*+1, −*z*; (iii) −*x*+1, −*y*+1, −*z*+1; (iv) *x*+1, *y*, *z*.

### 2-[2-(3-Nitrobenzenesulfonyl)hydrazinyl]-1,3-benzothiazole (II) Crystal data

**Table:**

C_13_H_10_N_4_O_4_S_2_	*Z* = 4
*M**_r_* = 350.37	*F*(000) = 720
Triclinic, *P*1	*D*_x_ = 1.639 Mg m^−^^3^
*a* = 6.83537 (15) Å	Mo *K*α radiation, λ = 0.71073 Å
*b* = 13.5788 (3) Å	Cell parameters from 18994 reflections
*c* = 15.6907 (4) Å	θ = 2.7–27.5°
α = 99.382 (2)°	µ = 0.40 mm^−^^1^
β = 98.2324 (19)°	*T* = 100 K
γ = 91.9841 (19)°	Plate, yellow
*V* = 1419.55 (6) Å^3^	0.15 × 0.10 × 0.03 mm

### 2-[2-(3-Nitrobenzenesulfonyl)hydrazinyl]-1,3-benzothiazole (II) Data collection

**Table:**

Rigaku Mercury CCD diffractometer	6033 reflections with *I* > 2σ(*I*)
ω scans	*R*_int_ = 0.018
Absorption correction: multi-scan (*FS_ABSCOR*; Rigaku, 2013)	θ_max_ = 27.5°, θ_min_ = 2.2°
*T*_min_ = 0.861, *T*_max_ = 1.000	*h* = −8→7
24032 measured reflections	*k* = −17→17
6465 independent reflections	*l* = −20→19

### 2-[2-(3-Nitrobenzenesulfonyl)hydrazinyl]-1,3-benzothiazole (II) Refinement

**Table:**

Refinement on *F*^2^	Primary atom site location: structure-invariant direct methods
Least-squares matrix: full	Hydrogen site location: mixed
*R*[*F*^2^ > 2σ(*F*^2^)] = 0.027	H atoms treated by a mixture of independent and constrained refinement
*wR*(*F*^2^) = 0.072	*w* = 1/[σ^2^(*F*_o_^2^) + (0.0369*P*)^2^ + 0.771*P*] where *P* = (*F*_o_^2^ + 2*F*_c_^2^)/3
*S* = 1.04	(Δ/σ)_max_ = 0.001
6465 reflections	Δρ_max_ = 0.40 e Å^−^^3^
427 parameters	Δρ_min_ = −0.38 e Å^−^^3^
0 restraints	

### 2-[2-(3-Nitrobenzenesulfonyl)hydrazinyl]-1,3-benzothiazole (II) Special details

**Table:**

Geometry. All esds (except the esd in the dihedral angle between two l.s. planes) are estimated using the full covariance matrix. The cell esds are taken into account individually in the estimation of esds in distances, angles and torsion angles; correlations between esds in cell parameters are only used when they are defined by crystal symmetry. An approximate (isotropic) treatment of cell esds is used for estimating esds involving l.s. planes.

### 2-[2-(3-Nitrobenzenesulfonyl)hydrazinyl]-1,3-benzothiazole (II) Fractional atomic coordinates and isotropic or equivalent isotropic displacement parameters (Å^2^)

**Table:**

	*x*	*y*	*z*	*U*_iso_*/*U*_eq_	
C1	0.43539 (18)	0.33184 (9)	0.47102 (8)	0.0141 (2)	
C2	0.55062 (19)	0.27184 (10)	0.51978 (8)	0.0169 (2)	
H3A	0.6190	0.2193	0.4915	0.020*	
C3	0.5642 (2)	0.28988 (10)	0.61023 (9)	0.0202 (3)	
H3B	0.6435	0.2497	0.6439	0.024*	
C4	0.4627 (2)	0.36625 (11)	0.65225 (9)	0.0210 (3)	
H4	0.4751	0.3779	0.7142	0.025*	
C5	0.3439 (2)	0.42538 (10)	0.60476 (8)	0.0185 (3)	
H5	0.2733	0.4767	0.6333	0.022*	
C6	0.33125 (18)	0.40728 (9)	0.51429 (8)	0.0149 (2)	
C7	0.28467 (18)	0.38771 (9)	0.35588 (8)	0.0139 (2)	
C8	−0.12349 (18)	0.31751 (9)	0.11243 (8)	0.0140 (2)	
C9	−0.13679 (18)	0.36196 (9)	0.03836 (8)	0.0147 (2)	
H9	−0.1521	0.4317	0.0415	0.018*	
C10	−0.12680 (18)	0.30065 (9)	−0.04025 (8)	0.0151 (2)	
C11	−0.10555 (19)	0.19861 (10)	−0.04774 (9)	0.0177 (3)	
H11	−0.1002	0.1586	−0.1029	0.021*	
C12	−0.09235 (19)	0.15654 (10)	0.02733 (9)	0.0177 (3)	
H12	−0.0772	0.0868	0.0238	0.021*	
C13	−0.10106 (18)	0.21541 (9)	0.10793 (8)	0.0161 (2)	
H13	−0.0918	0.1862	0.1593	0.019*	
S1	0.19123 (5)	0.46708 (2)	0.43815 (2)	0.01478 (7)	
S2	−0.14385 (4)	0.39379 (2)	0.21312 (2)	0.01401 (7)	
N1	0.40625 (15)	0.32218 (8)	0.38016 (7)	0.0139 (2)	
N2	0.23064 (17)	0.39617 (9)	0.27112 (7)	0.0172 (2)	
H2	0.284 (3)	0.3656 (13)	0.2323 (11)	0.021*	
N3	0.07391 (16)	0.45532 (8)	0.25127 (7)	0.0156 (2)	
H3	0.101 (2)	0.4983 (13)	0.2204 (11)	0.019*	
N4	−0.14030 (17)	0.34643 (8)	−0.11915 (7)	0.0180 (2)	
O1	−0.27389 (14)	0.47084 (7)	0.19593 (6)	0.01904 (19)	
O2	−0.17746 (14)	0.32943 (7)	0.27375 (6)	0.01830 (19)	
O3	−0.15792 (16)	0.43763 (7)	−0.11068 (6)	0.0248 (2)	
O4	−0.13227 (18)	0.29368 (8)	−0.18884 (6)	0.0306 (2)	
C14	0.38706 (17)	0.27186 (9)	0.04845 (8)	0.0135 (2)	
C15	0.37430 (18)	0.35578 (10)	0.00746 (8)	0.0162 (2)	
H15	0.3734	0.4208	0.0407	0.019*	
C16	0.36290 (19)	0.34242 (10)	−0.08280 (9)	0.0188 (3)	
H16	0.3550	0.3990	−0.1114	0.023*	
C17	0.3629 (2)	0.24711 (10)	−0.13217 (9)	0.0203 (3)	
H17	0.3535	0.2398	−0.1939	0.024*	
C18	0.3765 (2)	0.16275 (10)	−0.09252 (8)	0.0183 (3)	
H18	0.3766	0.0979	−0.1261	0.022*	
C19	0.38981 (18)	0.17643 (9)	−0.00197 (8)	0.0146 (2)	
C20	0.40680 (18)	0.18334 (9)	0.15515 (8)	0.0139 (2)	
C21	0.15315 (19)	0.07929 (9)	0.36629 (8)	0.0163 (2)	
C22	0.22077 (19)	0.04559 (10)	0.44344 (8)	0.0165 (2)	
H22	0.2967	−0.0117	0.4438	0.020*	
C23	0.17295 (19)	0.09898 (10)	0.51990 (8)	0.0175 (3)	
C24	0.0642 (2)	0.18347 (10)	0.52236 (9)	0.0205 (3)	
H24	0.0348	0.2185	0.5761	0.025*	
C25	−0.0005 (2)	0.21522 (10)	0.44401 (10)	0.0211 (3)	
H25	−0.0747	0.2731	0.4439	0.025*	
C26	0.0421 (2)	0.16336 (10)	0.36575 (9)	0.0190 (3)	
H26	−0.0042	0.1850	0.3122	0.023*	
S3	0.40564 (5)	0.08651 (2)	0.06631 (2)	0.01512 (7)	
S4	0.21030 (5)	0.01172 (2)	0.26734 (2)	0.01740 (8)	
N5	0.39616 (15)	0.27357 (8)	0.13792 (7)	0.0136 (2)	
N6	0.41743 (18)	0.16301 (8)	0.23735 (7)	0.0184 (2)	
H6	0.430 (2)	0.2085 (13)	0.2795 (12)	0.022*	
N7	0.42029 (17)	0.06345 (8)	0.24827 (7)	0.0170 (2)	
H7	0.520 (3)	0.0496 (12)	0.2847 (11)	0.020*	
N8	0.23973 (17)	0.06263 (9)	0.60183 (7)	0.0205 (2)	
O5	0.26067 (17)	−0.08604 (7)	0.28157 (6)	0.0248 (2)	
O6	0.05911 (15)	0.02700 (8)	0.19880 (6)	0.0242 (2)	
O7	0.32765 (15)	−0.01577 (8)	0.59683 (7)	0.0260 (2)	
O8	0.20339 (17)	0.10970 (9)	0.67020 (7)	0.0312 (2)	

### 2-[2-(3-Nitrobenzenesulfonyl)hydrazinyl]-1,3-benzothiazole (II) Atomic displacement parameters (Å^2^)

**Table:**

	*U*^11^	*U*^22^	*U*^33^	*U*^12^	*U*^13^	*U*^23^
C1	0.0141 (6)	0.0152 (6)	0.0123 (6)	−0.0026 (4)	0.0022 (4)	0.0005 (4)
C2	0.0156 (6)	0.0167 (6)	0.0180 (6)	0.0007 (5)	0.0022 (5)	0.0023 (5)
C3	0.0207 (6)	0.0218 (7)	0.0186 (6)	0.0001 (5)	0.0000 (5)	0.0076 (5)
C4	0.0248 (7)	0.0256 (7)	0.0121 (6)	−0.0011 (5)	0.0017 (5)	0.0035 (5)
C5	0.0211 (6)	0.0208 (6)	0.0131 (6)	0.0011 (5)	0.0044 (5)	0.0002 (5)
C6	0.0143 (6)	0.0166 (6)	0.0132 (6)	−0.0002 (4)	0.0011 (4)	0.0019 (5)
C7	0.0145 (6)	0.0142 (6)	0.0124 (6)	−0.0014 (4)	0.0039 (4)	−0.0006 (4)
C8	0.0135 (5)	0.0154 (6)	0.0120 (6)	0.0018 (4)	0.0007 (4)	0.0005 (4)
C9	0.0150 (6)	0.0146 (6)	0.0139 (6)	0.0004 (4)	0.0014 (4)	0.0016 (5)
C10	0.0142 (6)	0.0177 (6)	0.0130 (6)	−0.0006 (4)	0.0008 (4)	0.0025 (5)
C11	0.0160 (6)	0.0173 (6)	0.0175 (6)	−0.0001 (5)	0.0012 (5)	−0.0028 (5)
C12	0.0177 (6)	0.0131 (6)	0.0211 (6)	0.0012 (5)	0.0006 (5)	0.0011 (5)
C13	0.0152 (6)	0.0164 (6)	0.0166 (6)	0.0012 (5)	0.0001 (5)	0.0043 (5)
S1	0.01749 (15)	0.01571 (15)	0.01076 (14)	0.00315 (11)	0.00279 (11)	0.00015 (11)
S2	0.01745 (15)	0.01462 (14)	0.01033 (14)	0.00364 (11)	0.00230 (11)	0.00244 (11)
N1	0.0147 (5)	0.0151 (5)	0.0114 (5)	−0.0002 (4)	0.0026 (4)	0.0005 (4)
N2	0.0186 (5)	0.0224 (6)	0.0106 (5)	0.0069 (4)	0.0035 (4)	0.0004 (4)
N3	0.0193 (5)	0.0144 (5)	0.0130 (5)	0.0019 (4)	0.0015 (4)	0.0030 (4)
N4	0.0196 (5)	0.0212 (6)	0.0126 (5)	−0.0015 (4)	0.0015 (4)	0.0017 (4)
O1	0.0238 (5)	0.0196 (5)	0.0145 (4)	0.0093 (4)	0.0029 (4)	0.0032 (4)
O2	0.0222 (5)	0.0197 (5)	0.0144 (4)	0.0023 (4)	0.0039 (4)	0.0057 (4)
O3	0.0390 (6)	0.0192 (5)	0.0181 (5)	0.0015 (4)	0.0069 (4)	0.0060 (4)
O4	0.0487 (7)	0.0290 (6)	0.0116 (5)	0.0004 (5)	0.0035 (4)	−0.0022 (4)
C14	0.0113 (5)	0.0162 (6)	0.0131 (6)	0.0016 (4)	0.0024 (4)	0.0018 (5)
C15	0.0151 (6)	0.0158 (6)	0.0176 (6)	0.0016 (4)	0.0020 (5)	0.0033 (5)
C16	0.0187 (6)	0.0206 (6)	0.0186 (6)	0.0025 (5)	0.0018 (5)	0.0087 (5)
C17	0.0223 (7)	0.0265 (7)	0.0126 (6)	0.0026 (5)	0.0014 (5)	0.0050 (5)
C18	0.0226 (6)	0.0187 (6)	0.0136 (6)	0.0038 (5)	0.0031 (5)	0.0015 (5)
C19	0.0152 (6)	0.0151 (6)	0.0138 (6)	0.0025 (4)	0.0019 (4)	0.0036 (5)
C20	0.0145 (6)	0.0146 (6)	0.0121 (6)	0.0007 (4)	0.0030 (4)	0.0003 (4)
C21	0.0192 (6)	0.0151 (6)	0.0147 (6)	−0.0028 (5)	0.0037 (5)	0.0025 (5)
C22	0.0169 (6)	0.0154 (6)	0.0174 (6)	−0.0023 (5)	0.0026 (5)	0.0040 (5)
C23	0.0175 (6)	0.0196 (6)	0.0156 (6)	−0.0048 (5)	0.0026 (5)	0.0042 (5)
C24	0.0202 (6)	0.0195 (6)	0.0218 (7)	−0.0041 (5)	0.0082 (5)	0.0000 (5)
C25	0.0202 (6)	0.0161 (6)	0.0290 (7)	0.0001 (5)	0.0080 (5)	0.0058 (5)
C26	0.0197 (6)	0.0175 (6)	0.0212 (7)	−0.0008 (5)	0.0035 (5)	0.0078 (5)
S3	0.02245 (16)	0.01240 (14)	0.01072 (14)	0.00307 (11)	0.00390 (11)	0.00095 (11)
S4	0.02582 (17)	0.01316 (15)	0.01286 (15)	−0.00091 (12)	0.00193 (12)	0.00245 (11)
N5	0.0158 (5)	0.0131 (5)	0.0118 (5)	0.0008 (4)	0.0029 (4)	0.0009 (4)
N6	0.0332 (6)	0.0112 (5)	0.0108 (5)	−0.0002 (4)	0.0048 (4)	0.0009 (4)
N7	0.0238 (6)	0.0146 (5)	0.0135 (5)	0.0035 (4)	0.0034 (4)	0.0039 (4)
N8	0.0198 (5)	0.0257 (6)	0.0152 (5)	−0.0052 (5)	0.0021 (4)	0.0030 (4)
O5	0.0423 (6)	0.0128 (4)	0.0197 (5)	0.0008 (4)	0.0056 (4)	0.0035 (4)
O6	0.0283 (5)	0.0252 (5)	0.0171 (5)	−0.0024 (4)	−0.0020 (4)	0.0038 (4)
O7	0.0274 (5)	0.0324 (6)	0.0192 (5)	0.0041 (4)	0.0015 (4)	0.0090 (4)
O8	0.0406 (6)	0.0368 (6)	0.0148 (5)	−0.0036 (5)	0.0069 (4)	−0.0009 (4)

### 2-[2-(3-Nitrobenzenesulfonyl)hydrazinyl]-1,3-benzothiazole (II) Geometric parameters (Å, º)

**Table:**

C1—C2	1.3944 (18)	C14—N5	1.3927 (16)
C1—N1	1.3947 (16)	C14—C15	1.3963 (17)
C1—C6	1.4068 (17)	C14—C19	1.4061 (17)
C2—C3	1.3894 (19)	C15—C16	1.3887 (18)
C2—H3A	0.9500	C15—H15	0.9500
C3—C4	1.396 (2)	C16—C17	1.3958 (19)
C3—H3B	0.9500	C16—H16	0.9500
C4—C5	1.3877 (19)	C17—C18	1.3895 (19)
C4—H4	0.9500	C17—H17	0.9500
C5—C6	1.3902 (17)	C18—C19	1.3918 (18)
C5—H5	0.9500	C18—H18	0.9500
C6—S1	1.7450 (13)	C19—S3	1.7473 (13)
C7—N1	1.3033 (16)	C20—N5	1.2992 (16)
C7—N2	1.3529 (16)	C20—N6	1.3549 (16)
C7—S1	1.7511 (12)	C20—S3	1.7522 (12)
C8—C9	1.3876 (17)	C21—C22	1.3869 (18)
C8—C13	1.3917 (17)	C21—C26	1.3934 (18)
C8—S2	1.7685 (12)	C21—S4	1.7717 (13)
C9—C10	1.3829 (17)	C22—C23	1.3846 (18)
C9—H9	0.9500	C22—H22	0.9500
C10—C11	1.3858 (18)	C23—C24	1.3868 (19)
C10—N4	1.4661 (16)	C23—N8	1.4693 (17)
C11—C12	1.3837 (19)	C24—C25	1.387 (2)
C11—H11	0.9500	C24—H24	0.9500
C12—C13	1.3918 (18)	C25—C26	1.387 (2)
C12—H12	0.9500	C25—H25	0.9500
C13—H13	0.9500	C26—H26	0.9500
S2—O2	1.4287 (9)	S4—O5	1.4271 (10)
S2—O1	1.4301 (9)	S4—O6	1.4272 (10)
S2—N3	1.6636 (11)	S4—N7	1.6628 (12)
N2—N3	1.3912 (15)	N6—N7	1.3906 (15)
N2—H2	0.816 (18)	N6—H6	0.820 (18)
N3—H3	0.848 (17)	N7—H7	0.871 (17)
N4—O4	1.2150 (15)	N8—O8	1.2186 (16)
N4—O3	1.2358 (15)	N8—O7	1.2374 (16)
			
C2—C1—N1	125.63 (11)	N5—C14—C15	125.14 (11)
C2—C1—C6	119.30 (11)	N5—C14—C19	115.21 (11)
N1—C1—C6	115.02 (11)	C15—C14—C19	119.65 (11)
C3—C2—C1	119.12 (12)	C16—C15—C14	118.75 (12)
C3—C2—H3A	120.4	C16—C15—H15	120.6
C1—C2—H3A	120.4	C14—C15—H15	120.6
C2—C3—C4	120.83 (12)	C15—C16—C17	120.99 (12)
C2—C3—H3B	119.6	C15—C16—H16	119.5
C4—C3—H3B	119.6	C17—C16—H16	119.5
C5—C4—C3	120.91 (12)	C18—C17—C16	121.09 (12)
C5—C4—H4	119.5	C18—C17—H17	119.5
C3—C4—H4	119.5	C16—C17—H17	119.5
C4—C5—C6	118.08 (12)	C17—C18—C19	117.81 (12)
C4—C5—H5	121.0	C17—C18—H18	121.1
C6—C5—H5	121.0	C19—C18—H18	121.1
C5—C6—C1	121.74 (12)	C18—C19—C14	121.70 (12)
C5—C6—S1	128.32 (10)	C18—C19—S3	128.77 (10)
C1—C6—S1	109.93 (9)	C14—C19—S3	109.51 (9)
N1—C7—N2	122.68 (11)	N5—C20—N6	122.64 (11)
N1—C7—S1	117.31 (9)	N5—C20—S3	116.93 (9)
N2—C7—S1	120.01 (10)	N6—C20—S3	120.43 (9)
C9—C8—C13	121.44 (11)	C22—C21—C26	121.43 (12)
C9—C8—S2	118.05 (9)	C22—C21—S4	118.12 (10)
C13—C8—S2	120.47 (10)	C26—C21—S4	120.45 (10)
C10—C9—C8	117.29 (11)	C23—C22—C21	117.16 (12)
C10—C9—H9	121.4	C23—C22—H22	121.4
C8—C9—H9	121.4	C21—C22—H22	121.4
C9—C10—C11	123.15 (12)	C22—C23—C24	123.27 (12)
C9—C10—N4	118.01 (11)	C22—C23—N8	117.66 (12)
C11—C10—N4	118.84 (11)	C24—C23—N8	119.07 (12)
C12—C11—C10	118.18 (12)	C25—C24—C23	118.01 (13)
C12—C11—H11	120.9	C25—C24—H24	121.0
C10—C11—H11	120.9	C23—C24—H24	121.0
C11—C12—C13	120.65 (12)	C26—C25—C24	120.68 (13)
C11—C12—H12	119.7	C26—C25—H25	119.7
C13—C12—H12	119.7	C24—C25—H25	119.7
C8—C13—C12	119.28 (12)	C25—C26—C21	119.45 (12)
C8—C13—H13	120.4	C25—C26—H26	120.3
C12—C13—H13	120.4	C21—C26—H26	120.3
C6—S1—C7	88.04 (6)	C19—S3—C20	88.30 (6)
O2—S2—O1	121.70 (6)	O5—S4—O6	121.88 (6)
O2—S2—N3	106.58 (6)	O5—S4—N7	103.87 (6)
O1—S2—N3	103.87 (6)	O6—S4—N7	106.93 (6)
O2—S2—C8	107.73 (6)	O5—S4—C21	108.35 (6)
O1—S2—C8	108.20 (6)	O6—S4—C21	107.54 (6)
N3—S2—C8	108.09 (6)	N7—S4—C21	107.49 (6)
C7—N1—C1	109.69 (10)	C20—N5—C14	110.05 (10)
C7—N2—N3	117.74 (11)	C20—N6—N7	118.05 (11)
C7—N2—H2	121.7 (12)	C20—N6—H6	120.6 (12)
N3—N2—H2	120.5 (12)	N7—N6—H6	121.2 (12)
N2—N3—S2	115.55 (9)	N6—N7—S4	116.35 (9)
N2—N3—H3	113.4 (11)	N6—N7—H7	114.9 (11)
S2—N3—H3	112.1 (11)	S4—N7—H7	110.4 (11)
O4—N4—O3	123.45 (12)	O8—N8—O7	123.72 (12)
O4—N4—C10	118.93 (11)	O8—N8—C23	118.84 (12)
O3—N4—C10	117.61 (10)	O7—N8—C23	117.43 (11)
			
N1—C1—C2—C3	−179.34 (12)	N5—C14—C15—C16	−178.92 (12)
C6—C1—C2—C3	−1.78 (18)	C19—C14—C15—C16	0.54 (18)
C1—C2—C3—C4	0.6 (2)	C14—C15—C16—C17	0.38 (19)
C2—C3—C4—C5	0.7 (2)	C15—C16—C17—C18	−0.7 (2)
C3—C4—C5—C6	−0.9 (2)	C16—C17—C18—C19	0.1 (2)
C4—C5—C6—C1	−0.28 (19)	C17—C18—C19—C14	0.88 (19)
C4—C5—C6—S1	178.53 (10)	C17—C18—C19—S3	179.20 (10)
C2—C1—C6—C5	1.64 (19)	N5—C14—C19—C18	178.32 (11)
N1—C1—C6—C5	179.45 (11)	C15—C14—C19—C18	−1.20 (19)
C2—C1—C6—S1	−177.37 (10)	N5—C14—C19—S3	−0.30 (14)
N1—C1—C6—S1	0.44 (14)	C15—C14—C19—S3	−179.81 (9)
C13—C8—C9—C10	−0.02 (18)	C26—C21—C22—C23	0.08 (19)
S2—C8—C9—C10	−178.02 (9)	S4—C21—C22—C23	−179.45 (9)
C8—C9—C10—C11	0.36 (19)	C21—C22—C23—C24	−0.62 (19)
C8—C9—C10—N4	−179.90 (11)	C21—C22—C23—N8	178.59 (11)
C9—C10—C11—C12	−0.48 (19)	C22—C23—C24—C25	0.4 (2)
N4—C10—C11—C12	179.79 (11)	N8—C23—C24—C25	−178.77 (11)
C10—C11—C12—C13	0.25 (19)	C23—C24—C25—C26	0.3 (2)
C9—C8—C13—C12	−0.19 (19)	C24—C25—C26—C21	−0.8 (2)
S2—C8—C13—C12	177.77 (10)	C22—C21—C26—C25	0.63 (19)
C11—C12—C13—C8	0.07 (19)	S4—C21—C26—C25	−179.85 (10)
C5—C6—S1—C7	−179.42 (13)	C18—C19—S3—C20	−178.41 (13)
C1—C6—S1—C7	−0.50 (9)	C14—C19—S3—C20	0.08 (9)
N1—C7—S1—C6	0.52 (10)	N5—C20—S3—C19	0.17 (10)
N2—C7—S1—C6	−179.24 (11)	N6—C20—S3—C19	179.86 (11)
C9—C8—S2—O2	165.20 (10)	C22—C21—S4—O5	19.55 (12)
C13—C8—S2—O2	−12.82 (12)	C26—C21—S4—O5	−159.98 (11)
C9—C8—S2—O1	31.90 (12)	C22—C21—S4—O6	153.05 (10)
C13—C8—S2—O1	−146.12 (10)	C26—C21—S4—O6	−26.48 (12)
C9—C8—S2—N3	−79.99 (11)	C22—C21—S4—N7	−92.13 (11)
C13—C8—S2—N3	101.99 (11)	C26—C21—S4—N7	88.34 (11)
N2—C7—N1—C1	179.39 (11)	N6—C20—N5—C14	179.96 (12)
S1—C7—N1—C1	−0.35 (13)	S3—C20—N5—C14	−0.36 (14)
C2—C1—N1—C7	177.58 (12)	C15—C14—N5—C20	179.91 (12)
C6—C1—N1—C7	−0.07 (15)	C19—C14—N5—C20	0.42 (15)
N1—C7—N2—N3	168.50 (11)	N5—C20—N6—N7	177.46 (11)
S1—C7—N2—N3	−11.75 (16)	S3—C20—N6—N7	−2.20 (17)
C7—N2—N3—S2	−99.88 (12)	C20—N6—N7—S4	−103.52 (12)
O2—S2—N3—N2	52.10 (10)	O5—S4—N7—N6	−176.11 (9)
O1—S2—N3—N2	−178.24 (9)	O6—S4—N7—N6	53.82 (10)
C8—S2—N3—N2	−63.47 (10)	C21—S4—N7—N6	−61.41 (10)
C9—C10—N4—O4	−179.83 (12)	C22—C23—N8—O8	178.31 (12)
C11—C10—N4—O4	−0.08 (18)	C24—C23—N8—O8	−2.44 (18)
C9—C10—N4—O3	0.70 (17)	C22—C23—N8—O7	−2.85 (17)
C11—C10—N4—O3	−179.55 (12)	C24—C23—N8—O7	176.39 (12)

### 2-[2-(3-Nitrobenzenesulfonyl)hydrazinyl]-1,3-benzothiazole (II) Hydrogen-bond geometry (Å, º)

**Table:**

*D*—H···*A*	*D*—H	H···*A*	*D*···*A*	*D*—H···*A*
N2—H2···N5	0.816 (18)	2.033 (18)	2.8447 (15)	172.7 (16)
N3—H3···O3^i^	0.848 (17)	2.129 (18)	2.9427 (15)	160.8 (15)
N6—H6···N1	0.820 (18)	2.050 (18)	2.8601 (15)	169.2 (17)
N7—H7···O7^ii^	0.871 (17)	2.123 (18)	2.9472 (15)	157.6 (15)
C15—H15···O3^i^	0.95	2.65	3.4888 (17)	147
C26—H26···O2	0.95	2.44	3.1774 (16)	134
C5—H5···O1^iii^	0.95	2.66	3.3218 (16)	127
C13—H13···O6	0.95	2.56	3.2731 (16)	133

Symmetry codes: (i) −*x*, −*y*+1, −*z*; (ii) −*x*+1, −*y*, −*z*+1; (iii) −*x*, −*y*+1, −*z*+1.

### 2-[2-(3-Nitrobenzenesulfonyl)hydrazinyl]-1,3-benzothiazol-3-ium; 3-nitrobenzenesulfonate (III) Crystal data

**Table:**

C_13_H_11_N_4_O_4_S_2_^+^·C_6_H_4_NO_5_S^−^	*Z* = 2
*M**_r_* = 553.54	*F*(000) = 568
Triclinic, *P*1	*D*_x_ = 1.625 Mg m^−^^3^
*a* = 10.0399 (5) Å	Mo *K*α radiation, λ = 0.71073 Å
*b* = 10.7585 (4) Å	Cell parameters from 14254 reflections
*c* = 11.3372 (6) Å	θ = 2.0–27.5°
α = 85.607 (4)°	µ = 0.39 mm^−^^1^
β = 71.369 (5)°	*T* = 100 K
γ = 77.115 (4)°	Plate, colourless
*V* = 1131.16 (10) Å^3^	0.23 × 0.18 × 0.04 mm

### 2-[2-(3-Nitrobenzenesulfonyl)hydrazinyl]-1,3-benzothiazol-3-ium; 3-nitrobenzenesulfonate (III) Data collection

**Table:**

Rigaku Mercury CCD diffractometer	4880 reflections with *I* > 2σ(*I*)
ω scans	*R*_int_ = 0.017
Absorption correction: multi-scan (*FS_ABSCOR*; Rigaku, 2013)	θ_max_ = 27.6°, θ_min_ = 2.2°
*T*_min_ = 0.879, *T*_max_ = 1.000	*h* = −13→11
19781 measured reflections	*k* = −14→13
5159 independent reflections	*l* = −14→14

### 2-[2-(3-Nitrobenzenesulfonyl)hydrazinyl]-1,3-benzothiazol-3-ium; 3-nitrobenzenesulfonate (III) Refinement

**Table:**

Refinement on *F*^2^	Primary atom site location: structure-invariant direct methods
Least-squares matrix: full	Hydrogen site location: mixed
*R*[*F*^2^ > 2σ(*F*^2^)] = 0.029	H atoms treated by a mixture of independent and constrained refinement
*wR*(*F*^2^) = 0.076	*w* = 1/[σ^2^(*F*_o_^2^) + (0.0353*P*)^2^ + 0.8265*P*] where *P* = (*F*_o_^2^ + 2*F*_c_^2^)/3
*S* = 1.02	(Δ/σ)_max_ = 0.002
5159 reflections	Δρ_max_ = 0.39 e Å^−^^3^
334 parameters	Δρ_min_ = −0.42 e Å^−^^3^
0 restraints	

### 2-[2-(3-Nitrobenzenesulfonyl)hydrazinyl]-1,3-benzothiazol-3-ium; 3-nitrobenzenesulfonate (III) Special details

**Table:**

Geometry. All esds (except the esd in the dihedral angle between two l.s. planes) are estimated using the full covariance matrix. The cell esds are taken into account individually in the estimation of esds in distances, angles and torsion angles; correlations between esds in cell parameters are only used when they are defined by crystal symmetry. An approximate (isotropic) treatment of cell esds is used for estimating esds involving l.s. planes.

### 2-[2-(3-Nitrobenzenesulfonyl)hydrazinyl]-1,3-benzothiazol-3-ium; 3-nitrobenzenesulfonate (III) Fractional atomic coordinates and isotropic or equivalent isotropic displacement parameters (Å^2^)

**Table:**

	*x*	*y*	*z*	*U*_iso_*/*U*_eq_	
C1	1.07390 (14)	0.16015 (12)	−0.12063 (12)	0.0159 (3)	
C2	1.21341 (15)	0.10073 (13)	−0.18674 (14)	0.0198 (3)	
H2A	1.2930	0.1380	−0.1940	0.024*	
C3	1.23134 (15)	−0.01528 (13)	−0.24169 (14)	0.0211 (3)	
H3A	1.3252	−0.0581	−0.2878	0.025*	
C4	1.11437 (15)	−0.07065 (13)	−0.23068 (13)	0.0197 (3)	
H4	1.1304	−0.1505	−0.2689	0.024*	
C5	0.97546 (15)	−0.01107 (13)	−0.16489 (13)	0.0177 (3)	
H5	0.8958	−0.0484	−0.1574	0.021*	
C6	0.95732 (14)	0.10512 (12)	−0.11042 (12)	0.0151 (2)	
C7	0.84167 (14)	0.28675 (12)	−0.00034 (12)	0.0151 (2)	
C8	0.55707 (14)	0.47110 (13)	0.32289 (12)	0.0167 (3)	
C9	0.53199 (16)	0.36315 (14)	0.39425 (13)	0.0211 (3)	
H9	0.6099	0.3004	0.4059	0.025*	
C10	0.39098 (18)	0.34797 (15)	0.44861 (14)	0.0269 (3)	
H10	0.3725	0.2748	0.4983	0.032*	
C11	0.27802 (17)	0.43861 (17)	0.43063 (14)	0.0285 (3)	
H11	0.1816	0.4289	0.4679	0.034*	
C12	0.30799 (16)	0.54390 (16)	0.35725 (13)	0.0255 (3)	
C13	0.44587 (15)	0.56403 (14)	0.30272 (13)	0.0209 (3)	
H13	0.4637	0.6378	0.2538	0.025*	
S1	1.01665 (3)	0.30546 (3)	−0.04133 (3)	0.01753 (8)	
S2	0.73513 (3)	0.49153 (3)	0.25131 (3)	0.01507 (8)	
N1	0.82742 (12)	0.17982 (11)	−0.04048 (10)	0.0154 (2)	
H1	0.746 (2)	0.1638 (16)	−0.0270 (16)	0.018*	
N2	0.73035 (13)	0.37297 (11)	0.06510 (11)	0.0176 (2)	
H2	0.646 (2)	0.3709 (17)	0.0737 (16)	0.021*	
N3	0.76169 (12)	0.48236 (11)	0.09996 (11)	0.0166 (2)	
H3	0.7208 (19)	0.5525 (17)	0.0710 (16)	0.020*	
N4	0.18823 (15)	0.63839 (18)	0.33454 (13)	0.0396 (4)	
O1	0.82921 (11)	0.38488 (9)	0.28530 (10)	0.0220 (2)	
O2	0.74518 (11)	0.61876 (9)	0.26804 (9)	0.0195 (2)	
O3	0.21622 (15)	0.73477 (17)	0.27552 (14)	0.0584 (5)	
O4	0.06728 (14)	0.61654 (19)	0.37558 (15)	0.0589 (5)	
C14	0.31611 (14)	0.18627 (13)	0.18875 (13)	0.0164 (3)	
C15	0.36568 (15)	0.07109 (13)	0.23976 (13)	0.0197 (3)	
H15	0.4570	0.0192	0.1998	0.024*	
C16	0.27603 (16)	0.03482 (14)	0.35187 (14)	0.0218 (3)	
C17	0.14253 (15)	0.10749 (14)	0.41347 (13)	0.0219 (3)	
H17	0.0849	0.0799	0.4907	0.026*	
C18	0.09530 (15)	0.22141 (14)	0.35952 (14)	0.0212 (3)	
H18	0.0036	0.2727	0.3995	0.025*	
C19	0.18114 (15)	0.26111 (13)	0.24729 (13)	0.0194 (3)	
H19	0.1480	0.3392	0.2103	0.023*	
S3	0.42650 (3)	0.24195 (3)	0.04827 (3)	0.01634 (8)	
N5	0.32639 (15)	−0.08700 (13)	0.40760 (13)	0.0315 (3)	
O5	0.45472 (11)	0.36044 (9)	0.08076 (10)	0.0225 (2)	
O6	0.55538 (10)	0.14188 (9)	0.00769 (9)	0.0200 (2)	
O7	0.34166 (11)	0.26546 (10)	−0.03675 (10)	0.0224 (2)	
O8	0.23692 (14)	−0.13450 (12)	0.48774 (11)	0.0393 (3)	
O9	0.45499 (14)	−0.13511 (13)	0.37160 (14)	0.0493 (4)	

### 2-[2-(3-Nitrobenzenesulfonyl)hydrazinyl]-1,3-benzothiazol-3-ium; 3-nitrobenzenesulfonate (III) Atomic displacement parameters (Å^2^)

**Table:**

	*U*^11^	*U*^22^	*U*^33^	*U*^12^	*U*^13^	*U*^23^
C1	0.0170 (6)	0.0135 (6)	0.0176 (6)	−0.0045 (5)	−0.0045 (5)	−0.0013 (5)
C2	0.0157 (6)	0.0183 (6)	0.0247 (7)	−0.0044 (5)	−0.0044 (5)	−0.0016 (5)
C3	0.0179 (7)	0.0176 (6)	0.0247 (7)	−0.0003 (5)	−0.0041 (5)	−0.0034 (5)
C4	0.0241 (7)	0.0141 (6)	0.0214 (7)	−0.0027 (5)	−0.0081 (5)	−0.0031 (5)
C5	0.0202 (6)	0.0163 (6)	0.0195 (6)	−0.0065 (5)	−0.0082 (5)	−0.0002 (5)
C6	0.0149 (6)	0.0156 (6)	0.0149 (6)	−0.0037 (5)	−0.0048 (5)	0.0008 (5)
C7	0.0146 (6)	0.0172 (6)	0.0142 (6)	−0.0054 (5)	−0.0040 (5)	0.0006 (5)
C8	0.0162 (6)	0.0195 (6)	0.0141 (6)	−0.0047 (5)	−0.0032 (5)	−0.0019 (5)
C9	0.0269 (7)	0.0191 (7)	0.0171 (6)	−0.0079 (5)	−0.0041 (5)	−0.0012 (5)
C10	0.0321 (8)	0.0294 (8)	0.0193 (7)	−0.0163 (6)	−0.0011 (6)	−0.0025 (6)
C11	0.0225 (7)	0.0459 (9)	0.0186 (7)	−0.0158 (7)	−0.0005 (6)	−0.0091 (6)
C12	0.0178 (7)	0.0423 (9)	0.0155 (6)	−0.0017 (6)	−0.0056 (5)	−0.0059 (6)
C13	0.0193 (7)	0.0270 (7)	0.0143 (6)	−0.0020 (5)	−0.0042 (5)	−0.0004 (5)
S1	0.01279 (15)	0.01565 (15)	0.02306 (17)	−0.00532 (12)	−0.00137 (12)	−0.00507 (12)
S2	0.01461 (15)	0.01342 (15)	0.01680 (16)	−0.00269 (11)	−0.00443 (12)	−0.00064 (11)
N1	0.0128 (5)	0.0175 (5)	0.0167 (5)	−0.0055 (4)	−0.0039 (4)	−0.0018 (4)
N2	0.0124 (5)	0.0190 (6)	0.0214 (6)	−0.0053 (4)	−0.0025 (4)	−0.0063 (4)
N3	0.0174 (5)	0.0146 (5)	0.0174 (5)	−0.0046 (4)	−0.0036 (4)	−0.0021 (4)
N4	0.0208 (7)	0.0723 (12)	0.0192 (7)	0.0057 (7)	−0.0070 (5)	−0.0037 (7)
O1	0.0195 (5)	0.0190 (5)	0.0278 (5)	−0.0004 (4)	−0.0105 (4)	0.0006 (4)
O2	0.0224 (5)	0.0160 (5)	0.0196 (5)	−0.0059 (4)	−0.0044 (4)	−0.0021 (4)
O3	0.0337 (7)	0.0828 (12)	0.0411 (8)	0.0156 (7)	−0.0114 (6)	0.0212 (8)
O4	0.0166 (6)	0.1043 (14)	0.0500 (9)	−0.0015 (7)	−0.0084 (6)	−0.0081 (9)
C14	0.0150 (6)	0.0186 (6)	0.0185 (6)	−0.0065 (5)	−0.0071 (5)	0.0009 (5)
C15	0.0154 (6)	0.0201 (7)	0.0225 (7)	−0.0030 (5)	−0.0053 (5)	0.0020 (5)
C16	0.0209 (7)	0.0207 (7)	0.0222 (7)	−0.0030 (5)	−0.0066 (5)	0.0050 (5)
C17	0.0199 (7)	0.0261 (7)	0.0187 (7)	−0.0061 (6)	−0.0037 (5)	−0.0005 (5)
C18	0.0161 (6)	0.0239 (7)	0.0228 (7)	−0.0015 (5)	−0.0056 (5)	−0.0054 (5)
C19	0.0197 (7)	0.0174 (6)	0.0233 (7)	−0.0034 (5)	−0.0099 (5)	−0.0010 (5)
S3	0.01426 (15)	0.01613 (16)	0.02054 (17)	−0.00592 (12)	−0.00693 (12)	0.00324 (12)
N5	0.0284 (7)	0.0281 (7)	0.0269 (7)	0.0010 (5)	−0.0006 (5)	0.0102 (5)
O5	0.0195 (5)	0.0176 (5)	0.0322 (6)	−0.0076 (4)	−0.0083 (4)	0.0000 (4)
O6	0.0152 (5)	0.0189 (5)	0.0253 (5)	−0.0058 (4)	−0.0043 (4)	0.0014 (4)
O7	0.0230 (5)	0.0253 (5)	0.0236 (5)	−0.0090 (4)	−0.0126 (4)	0.0066 (4)
O8	0.0388 (7)	0.0325 (6)	0.0298 (6)	−0.0029 (5)	0.0058 (5)	0.0143 (5)
O9	0.0287 (7)	0.0444 (8)	0.0511 (8)	0.0100 (6)	0.0022 (6)	0.0252 (6)

### 2-[2-(3-Nitrobenzenesulfonyl)hydrazinyl]-1,3-benzothiazol-3-ium; 3-nitrobenzenesulfonate (III) Geometric parameters (Å, º)

**Table:**

C1—C2	1.3913 (19)	S2—O2	1.4260 (10)
C1—C6	1.3950 (18)	S2—O1	1.4279 (10)
C1—S1	1.7513 (13)	S2—N3	1.6574 (12)
C2—C3	1.3876 (19)	N1—H1	0.841 (19)
C2—H2A	0.9500	N2—N3	1.3985 (16)
C3—C4	1.399 (2)	N2—H2	0.826 (19)
C3—H3A	0.9500	N3—H3	0.869 (18)
C4—C5	1.386 (2)	N4—O4	1.223 (2)
C4—H4	0.9500	N4—O3	1.227 (2)
C5—C6	1.3863 (18)	C14—C15	1.3844 (19)
C5—H5	0.9500	C14—C19	1.3949 (19)
C6—N1	1.3946 (17)	C14—S3	1.7736 (14)
C7—N1	1.3215 (17)	C15—C16	1.3884 (19)
C7—N2	1.3317 (17)	C15—H15	0.9500
C7—S1	1.7218 (13)	C16—C17	1.383 (2)
C8—C9	1.3867 (19)	C16—N5	1.4677 (18)
C8—C13	1.3887 (19)	C17—C18	1.383 (2)
C8—S2	1.7674 (14)	C17—H17	0.9500
C9—C10	1.393 (2)	C18—C19	1.387 (2)
C9—H9	0.9500	C18—H18	0.9500
C10—C11	1.379 (2)	C19—H19	0.9500
C10—H10	0.9500	S3—O7	1.4526 (10)
C11—C12	1.383 (2)	S3—O6	1.4558 (10)
C11—H11	0.9500	S3—O5	1.4630 (10)
C12—C13	1.383 (2)	N5—O9	1.2244 (18)
C12—N4	1.469 (2)	N5—O8	1.2270 (17)
C13—H13	0.9500		
			
C2—C1—C6	121.20 (12)	O2—S2—C8	110.40 (6)
C2—C1—S1	128.05 (10)	O1—S2—C8	107.66 (6)
C6—C1—S1	110.75 (10)	N3—S2—C8	104.82 (6)
C3—C2—C1	117.18 (13)	C7—N1—C6	113.48 (11)
C3—C2—H2A	121.4	C7—N1—H1	120.7 (12)
C1—C2—H2A	121.4	C6—N1—H1	125.7 (12)
C2—C3—C4	121.52 (13)	C7—N2—N3	116.58 (11)
C2—C3—H3A	119.2	C7—N2—H2	123.0 (12)
C4—C3—H3A	119.2	N3—N2—H2	119.4 (12)
C5—C4—C3	121.15 (13)	N2—N3—S2	114.02 (9)
C5—C4—H4	119.4	N2—N3—H3	113.4 (12)
C3—C4—H4	119.4	S2—N3—H3	111.7 (12)
C6—C5—C4	117.39 (12)	O4—N4—O3	124.11 (16)
C6—C5—H5	121.3	O4—N4—C12	118.27 (17)
C4—C5—H5	121.3	O3—N4—C12	117.62 (15)
C5—C6—N1	126.36 (12)	C15—C14—C19	121.07 (13)
C5—C6—C1	121.56 (12)	C15—C14—S3	119.86 (11)
N1—C6—C1	112.08 (12)	C19—C14—S3	119.06 (10)
N1—C7—N2	122.94 (12)	C14—C15—C16	117.05 (13)
N1—C7—S1	114.10 (10)	C14—C15—H15	121.5
N2—C7—S1	122.96 (10)	C16—C15—H15	121.5
C9—C8—C13	121.98 (13)	C17—C16—C15	123.45 (13)
C9—C8—S2	119.96 (11)	C17—C16—N5	118.58 (13)
C13—C8—S2	118.04 (11)	C15—C16—N5	117.97 (13)
C8—C9—C10	119.11 (14)	C18—C17—C16	118.15 (13)
C8—C9—H9	120.4	C18—C17—H17	120.9
C10—C9—H9	120.4	C16—C17—H17	120.9
C11—C10—C9	120.37 (14)	C17—C18—C19	120.32 (13)
C11—C10—H10	119.8	C17—C18—H18	119.8
C9—C10—H10	119.8	C19—C18—H18	119.8
C10—C11—C12	118.62 (14)	C18—C19—C14	119.95 (13)
C10—C11—H11	120.7	C18—C19—H19	120.0
C12—C11—H11	120.7	C14—C19—H19	120.0
C13—C12—C11	123.14 (15)	O7—S3—O6	113.88 (6)
C13—C12—N4	118.14 (15)	O7—S3—O5	111.37 (6)
C11—C12—N4	118.71 (14)	O6—S3—O5	113.23 (6)
C12—C13—C8	116.76 (14)	O7—S3—C14	105.96 (6)
C12—C13—H13	121.6	O6—S3—C14	106.14 (6)
C8—C13—H13	121.6	O5—S3—C14	105.46 (6)
C7—S1—C1	89.58 (6)	O9—N5—O8	123.89 (14)
O2—S2—O1	120.92 (6)	O9—N5—C16	118.16 (13)
O2—S2—N3	104.08 (6)	O8—N5—C16	117.95 (13)
O1—S2—N3	107.77 (6)		
			
C6—C1—C2—C3	−0.1 (2)	C5—C6—N1—C7	−179.60 (13)
S1—C1—C2—C3	179.86 (11)	C1—C6—N1—C7	0.83 (16)
C1—C2—C3—C4	−0.3 (2)	N1—C7—N2—N3	−179.22 (12)
C2—C3—C4—C5	0.4 (2)	S1—C7—N2—N3	0.16 (17)
C3—C4—C5—C6	−0.1 (2)	C7—N2—N3—S2	−110.54 (12)
C4—C5—C6—N1	−179.80 (13)	O2—S2—N3—N2	−168.76 (9)
C4—C5—C6—C1	−0.3 (2)	O1—S2—N3—N2	61.70 (11)
C2—C1—C6—C5	0.4 (2)	C8—S2—N3—N2	−52.78 (11)
S1—C1—C6—C5	−179.57 (10)	C13—C12—N4—O4	175.11 (15)
C2—C1—C6—N1	179.98 (12)	C11—C12—N4—O4	−4.1 (2)
S1—C1—C6—N1	0.03 (14)	C13—C12—N4—O3	−5.0 (2)
C13—C8—C9—C10	−0.8 (2)	C11—C12—N4—O3	175.70 (16)
S2—C8—C9—C10	−179.19 (11)	C19—C14—C15—C16	−0.8 (2)
C8—C9—C10—C11	0.6 (2)	S3—C14—C15—C16	177.97 (11)
C9—C10—C11—C12	0.3 (2)	C14—C15—C16—C17	−0.2 (2)
C10—C11—C12—C13	−1.2 (2)	C14—C15—C16—N5	179.96 (13)
C10—C11—C12—N4	177.97 (14)	C15—C16—C17—C18	0.9 (2)
C11—C12—C13—C8	1.1 (2)	N5—C16—C17—C18	−179.26 (14)
N4—C12—C13—C8	−178.12 (13)	C16—C17—C18—C19	−0.6 (2)
C9—C8—C13—C12	−0.1 (2)	C17—C18—C19—C14	−0.4 (2)
S2—C8—C13—C12	178.39 (11)	C15—C14—C19—C18	1.1 (2)
N1—C7—S1—C1	1.13 (11)	S3—C14—C19—C18	−177.70 (11)
N2—C7—S1—C1	−178.31 (12)	C15—C14—S3—O7	125.09 (11)
C2—C1—S1—C7	179.43 (14)	C19—C14—S3—O7	−56.11 (12)
C6—C1—S1—C7	−0.62 (10)	C15—C14—S3—O6	3.69 (13)
C9—C8—S2—O2	−135.32 (11)	C19—C14—S3—O6	−177.51 (10)
C13—C8—S2—O2	46.19 (13)	C15—C14—S3—O5	−116.72 (12)
C9—C8—S2—O1	−1.39 (13)	C19—C14—S3—O5	62.08 (12)
C13—C8—S2—O1	−179.89 (11)	C17—C16—N5—O9	−162.03 (16)
C9—C8—S2—N3	113.16 (12)	C15—C16—N5—O9	17.8 (2)
C13—C8—S2—N3	−65.33 (12)	C17—C16—N5—O8	17.9 (2)
N2—C7—N1—C6	178.09 (12)	C15—C16—N5—O8	−162.28 (15)
S1—C7—N1—C6	−1.34 (15)		

### 2-[2-(3-Nitrobenzenesulfonyl)hydrazinyl]-1,3-benzothiazol-3-ium; 3-nitrobenzenesulfonate (III) Hydrogen-bond geometry (Å, º)

**Table:**

*D*—H···*A*	*D*—H	H···*A*	*D*···*A*	*D*—H···*A*
N1—H1···O6	0.841 (19)	1.888 (19)	2.7267 (15)	175.2 (17)
N2—H2···O5	0.826 (19)	1.92 (2)	2.7489 (16)	175.4 (18)
N3—H3···O7^i^	0.869 (18)	1.968 (19)	2.8058 (16)	161.6 (16)
C2—H2*A*···O7^ii^	0.95	2.55	3.2510 (18)	130
C9—H9···O8^iii^	0.95	2.58	3.487 (2)	161

Symmetry codes: (i) −*x*+1, −*y*+1, −*z*; (ii) *x*+1, *y*, *z*; (iii) −*x*+1, −*y*, −*z*+1.
